# The Insurance of Nurses: A Hospital Chairman's Suggestion

**Published:** 1912-06-29

**Authors:** 


*To the Editor of* The Hospital.

### The Insurance of Nurses: A Hospital Chairman's Suggestion.

*To the Editor of* The Hospital.

Sir,—We have had a meeting of sisters and nurses at the hospital to discuss the best thing to be done when the Insurance Act comes into force. The sisters and nurses showed very great interest, and a great number of questions were asked.

It occurred to me as a result of thoughts on the meeting that if the hospital authorities and nurses were to bring pressure to bear on their members of Parliament when considerations for amending the Act come forward, a good suggestion would be that hospital nurses should have special treatment in this respect. They are, as a rule, very healthy owing to the conditions of their lives and the selection that is exercised in giving them employment. When ill they have the benefit of medical attendance and treatment, drugs, etc., in the hospital. Under the Act, according to their treatment, the 7s. 6d. will be devoted to their dependants. There is no crying need for this. In the event of their having no dependants, they may decide to whom it is given. If it is given to the hospital it will be difficult for the Committee to decide how to apply it.

If the Act were amended so as to give them unemployment benefits, it seems to me this would be of very much more value to them, as the age at which a hospital nurse can obtain employment is a comparatively young one—too old at forty applies more to this profession perhaps than to any other, excepting the theatrical. I think I might say without fear of contradiction that they are all unanimously against compulsory payment for sick insurance; but I think a scheme for unemployment benefits would appeal to them very much more. They would all be willing to help their sisters when out of work; and if it were explained to them that under the Act they are doing so by their own contributions and laying up for themselves in case of unemployment, it would appeal to a great majority of them, I think.

There is no doubt in my mind that under the Act as it at present exists the sisters and nurses will benefit best by joining an "approved" society; and I think it would be a very good course for The Hospital to take in naming "approved" societies which have been personally investigated by The Hospital as best for particular districts. Another scheme which I think would be very much approved by hospital nurses is such a one as is granted under the women's section of the "Hearts of Oak" Society: for a contribution of 3s. 3d. a quarter a bonus is given after ten years' membership of £7, after twenty years' membership of £16, and after thirty years' membership of £28, or at death or in special circumstances the net balance standing to the credit of the member will be paid.

All wanted to know if they never drew sick pay would they get their money back. If a scheme were introduced by which they got some bonus (however small) for not drawing sick pay, it would appeal to them.—Yours faithfully,

Hospital Chairman.

[We can recommend one Approved Society—namely, "The Nurses' Insurance Society," which is a separate section of the Royal National Pension Fund for Nurses. Its address is 15 Buckingham Street, Strand, W.C. As to the other points raised by "Hospital Chairman," we refer him to the article which appears under our Insurance Section this week.—Ed. The Hospital.]

